# Clozapine Triggering Cecal Volvulus in a Patient With Malrotation and Schizophrenia: Side Effect That Needs Emphasis

**DOI:** 10.7759/cureus.7971

**Published:** 2020-05-05

**Authors:** Jitender Aneja, Vaibhav Varshney, Sreesanth KS, Kartik Singhai, Taruna Yadav

**Affiliations:** 1 Psychiatry, All India Institute of Medical Sciences, Bathinda, IND; 2 Psychiatry, All India Institute of Medical Sciences, Jodhpur, IND; 3 Surgical Gastroenterology, All India Institute of Medical Sciences, Jodhpur, IND; 4 Radiology, All India Institute of Medical Sciences, Jodhpur, IND

**Keywords:** clozapine, hypomotility, cecal volvulus, constipation, hemicolectomy, adverse event

## Abstract

Clozapine induced gastrointestinal hypomotility (CIGH) has been suggested as one of the most common causes of significant morbidity and mortality. It commonly presents as constipation, but the reports of other small or large intestinal complications with fatal outcomes are not uncommon.

Hereby, we report the case of a 24-year-old male, suffering from schizophrenia and being managed with clozapine, who developed symptoms of acute intestinal obstruction due to ceco-colic volvulus. Subsequently, he was found to have intestinal malrotation at emergency laparotomy and underwent de-rotation of cecal volvulus and right hemicolectomy. He did well in the postoperative period and afterwards.

Cecal volvulus which in itself is a rare cause of intestinal obstruction in adults has not been observed consequent to clozapine treatment and required surgical therapy.

## Introduction

Cecal volvulus is a rare cause of intestinal obstruction and accounts for one third of all colonic volvulus. It ensues due to poorly fixed cecum and ascending colon to posterior peritoneum [1]. Although failure of retroperitoneal fixation occurs in 10%-15% of population, occurrence of cecal volvulus is rare suggesting requirement of some precipitating factors like constipation, postoperative paralytic ileus, pregnancy, etc. [2].

Clozapine, a second-generation antipsychotic, commonly prescribed for treatment-resistant schizophrenia (TRS) is also associated with a range of serious adverse effects. Agranulocytosis and cardiac adverse effects of clozapine have grabbed a lot of attention and guidelines. But little attention has been paid to clozapine induced gastrointestinal hypomotility (CIGH). Recent evidence has shown that mortality related to CIGH is up to 10-12 per 10,000 and that the rates were three times higher when compared with agranulocytosis [3]. Despite this, the gastrointestinal adverse effects of clozapine have largely been under-diagnosed and thus, often insufficiently managed, with no proper guidelines till date [4]. CIGH, commonly presents as constipation, but the reports of fecal impaction, paralytic ileus, intestinal obstruction, intestinal ischemia, bowel perforation, and fatal outcomes are not infrequent [5-6]. Although varying clinical presentations of CIGH are found in literature, to date there is no report of intestinal obstruction in the form of ceco-colic volvulus with malrotation in a patient with schizophrenia receiving clozapine therapy. Herein, we report the case of a young patient with schizophrenia who was on clozapine therapy and developed ceco-colic volvulus.

## Case presentation

A 24-year-old male was suffering from schizophrenia with features of Capgras syndrome and tobacco dependence syndrome for the last eight years and was under psychiatry treatment at our institute for a year. He was treated as inpatient and at the time of admission his mental state examination showed restricted and inappropriate affect, delusion of misidentification and doubles, as well as delusion of persecution, impaired abstraction, and judgement. The symptoms severity was rated on Positive and Negative Syndrome Scale (PANSS) that revealed a score of 87 [7]. In view of failed trials of olanzapine, haloperidol, and risperidone as well as nine sessions of modified bilateral electroconvulsive therapy, he was diagnosed with TRS and was started on clozapine (Sizopin, Sun Pharmaceuticals Industries Ltd., India). In the first three weeks of inpatient treatment, the dose of clozapine was built up to 250 mg per day. As per the treatment guidelines, monitoring for agranulocytosis and repeat electrocardiograms was done [8]. The dose of clozapine was increased slowly in view of excessive sedation. However, due to poor response, the dose of clozapine was further increased to up to 500 mg per day over the weeksfour to six of inpatient treatment. The PANSS was only reduced to 65 with this treatment, and therefore, trifluoperazine was added and increased up to 20 mg per day in the next two weeks. Dietary modifications and osmotic laxatives were advised for constipation, but patient would be nonadherent to this advice. In view of residual Capgras syndrome and negative symptoms, cognitive behavior therapy (CBT) was initiated. The patient was discharged after eight weeks of inpatient treatment and CBT was continued on outpatient basis. He followed up regularly with us for the next four weeks. But in the sixth week postdischarge, he presented to the surgical emergency with complaints of colicky pain in abdomen associated with recurrent bilious vomiting since a day. He also had abdominal distension with obstipation. Apart from this, there was no history of fever, trauma, or any other surgical intervention in the past. On physical examination he was dehydrated with tachycardia and distended abdomen with no peritoneal signs. The treating team ordered a complete hemogram, blood biochemistry, which was within normal limits.

**Figure 1:**
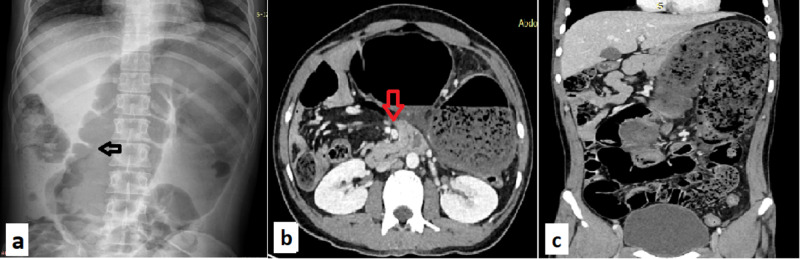
Abdominal radiograph and CECT abdomen. (a) Supine view showing dilated bowel loops in omega conformation with apex in left upper quadrant and haustration (arrow); (b-c) showing ceco-colic volvulus with transportation of mesenteric vessels (arrow). CECT, contrast enhanced computed tomography

Abdominal radiograph showed dilated bowel loops in omega conformation with apex in left upper quadrant and maintained haustra suggestive of cecal volvulus (Figure 1a). A contrast enhanced computed tomography (CECT) scan of the abdomen showed ceco-colic volvulus with malrotation of gut and edematous bowel without evidence of gangrene or perforation (Figure 1b,c).

**Figure 2:**
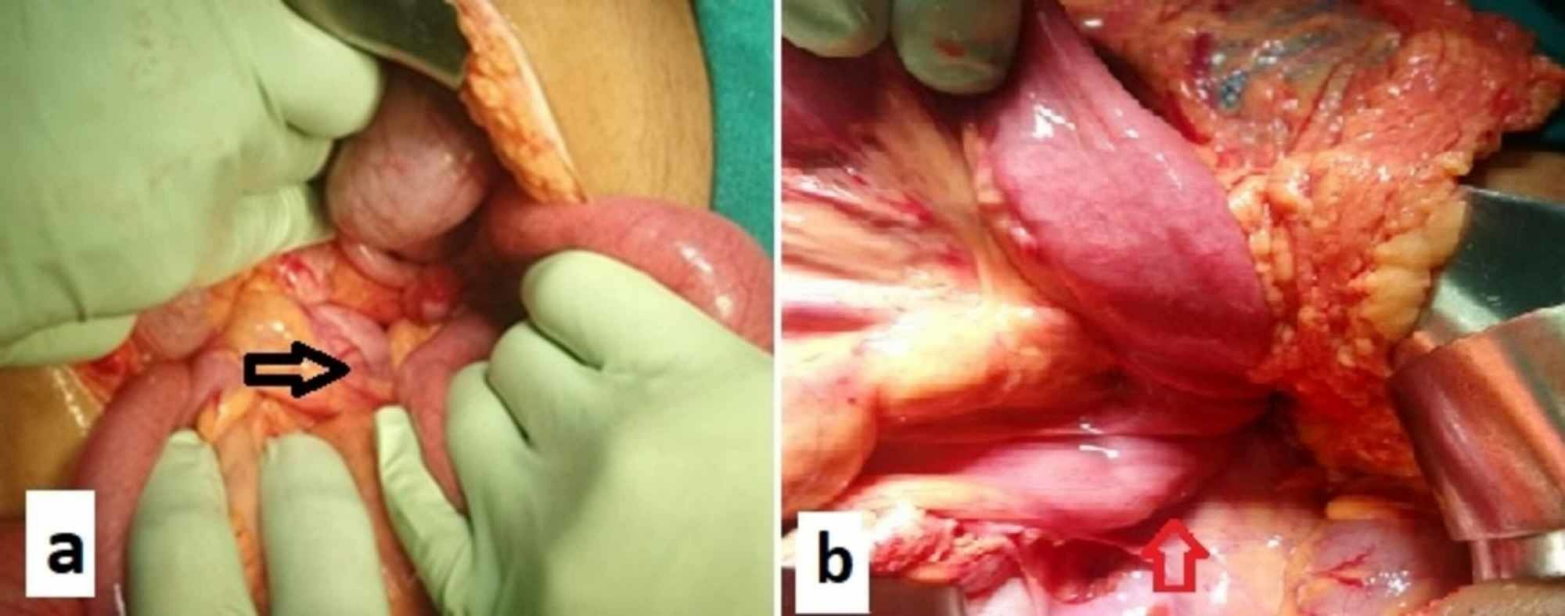
Intraoperative images. (a) Twisted cecum and ascending colon (arrow); (b) duodeno-jejunal flexure on the right side (arrow) suggestive of malrotation.

After an informed consent, he was taken for an emergency laparotomy. His cecum and ascending colon were hugely dilated with no evidence of frank gangrenous changes. As the cecal volvulus was de-rotated, cecum with ascending colon mesentery was found free from retroperitoneal attachment with long mesocolon and duodeno-jejunal flexure was found on the right side of midline. In view of friable bowel wall with doubtful viability, right hemicolectomy with ileo-transverse anastomosis was performed (Figure 2a,b).

Postoperatively, he was allowed orally on second postoperative day (POD) which he tolerated well. He developed paralytic ileus on fourth POD which was managed conservatively. He was discharged within a week of admission.

At the time of discharge, he was prescribed aripiprazole 2.5 mg/day. The dose of aripiprazole was built up to 10 mg/day over next four weeks and he did not develop any constipation, vomiting, or pain in abdomen. In view of worsening of psychotic symptoms, dose of aripiprazole was further increased to up to 20 mg/day. In view of patient refusal as well as lack of sufficient evidence of usefulness of clozapine re-challenge in cases of CIGH, this option was not pursued. Further, patient and his relatives were also educated about the necessary dietary modifications required to prevent recurrence of gastrointestinal symptoms.

## Discussion

Cecal volvulus, itself is a rare ailment which accounts for 1%-2% of all cases of intestinal obstruction in adults [9]. The disease is more prevalent in those aged 40-60 years with slight female predominance [10]. The prime predisposing factor is the improper fusion of cecal and colonic mesentery with retroperitoneum which lead to its rotation on axial plane. Despite this anatomic predilection which occurs in 10%-15% population, there seems a requirement of various aggravating factors like chronic constipation, adynamic ileus, adhesions/band, recent surgical manipulation, and pregnancy linked with it. Adulthood prevalence of malrotation is estimated to be 0.2%-0.5% and incidence of cecal volvulus in adult with malrotation is still rarer [2].

 Clozapine has been recommended as an effective evidence-based treatment for TRS (NICE, 2014). Clozapine leads to CIGH and constipation due to its anti-muscarinic, anti-serotonergic as well as anti-adrenergic properties which induce varying effects on gastrointestinal system [11]. Also, a co-prescription of medicines with anticholinergic properties namely tricyclic antidepressants, anticholinergic agents, first generation antipsychotics, opioid analgesics further increase the risk. There have been some case reports of severe CIGH that required surgery [12-13]. Further, Palmer et al. had analyzed severe cases of CIGH from medical and pharmacovigilance database in which the mortality rates to the extent of 18%-27.5% were reported [3-4]. But the obstruction of large intestine in form of development of ceco-volvulus is not reported till date.

In the case described here, though cecal volvulus is associated with malrotation in adults, exact incidence is not known due to rarity of disease. The CIGH associated with clozapine could have increased the risk of cecal volvulus in this patient who already has a predisposing factor in the form of intestinal malrotation. The risk factors for CIGH and subsequently intestinal obstruction included a high dose of clozapine, first four months of treatment, and male gender. Furthermore, lack of dietary modifications, minimal physical activity, and a co-prescription of trifluoperazine could have been other predisposing factors for the occurrence of CIGH. With best of our efforts, we could find only two case reports of an association of paralytic ileus with trifluoperazine [14-15]. Therefore, we believe that clozapine was the most likely precipitating factor for the ceco-colic volvulus in the index case.

Diagnosis of cecal volvulus is usually clinched with radiological imaging. Dilated cecum and colon resembling either coffee bean or omega loop with presence of haustra, apex towards left quadrant, one or two air-fluid level and absence of gas in rest of the colon are peculiar features of colonic volvulus [16]. However, CECT abdomen is the best modality for diagnosis and related complications. Distended cecum and ascending colon with a whirl sign are diagnostic of colonic volvulus, while transposition of superior mesenteric vessels, position of DJ flexure on midline or right side of midline and small bowel of right side are suggestive of malrotation [2]. The axial torsion leads to strangulation of vessels that causes gangrenous changes in the bowel. Hence, prompt diagnosis and surgical intervention is always necessary due to high incidence of morbidity and mortality. Nonoperative management like colonoscopy-guided reduction is not usually done due to its low success rate and associated risk of bowel perforation. Primary resection and anastomosis is associated with low morbidity, minimal recurrence, and best long-term outcome [2,17]. Other surgical options like cecopexy and cecostomy should be avoided as they are associated with high morbidity and recurrence [18].

## Conclusions

In conclusion, we emphasize that clozapine induced constipation may lead to severe complications. Therefore, suitable measures should be taken to firstly inform and educate the mental health professionals to recognize and prevent or manage the symptoms of CIGH. Secondly, this case highlights the importance of high index of suspicion and appropriate preoperative imaging and timely management of cecal volvulus to avoid major morbidity and mortality.
